# The effects of whole‐body vibration amplitude on glucose metabolism, inflammation, and skeletal muscle oxygenation

**DOI:** 10.14814/phy2.15208

**Published:** 2022-03-03

**Authors:** Adeola A. Sanni, Anson M. Blanks, Cassandra C. Derella, Chase Horsager, Reva H. Crandall, Jacob Looney, Savanna Sanchez, Kimberly Norland, Bingwei Ye, Jeffrey Thomas, Xiaoling Wang, Ryan A. Harris

**Affiliations:** ^1^ Department of Medicine Georgia Prevention Institute Augusta University Augusta Georgia USA; ^2^ Sport and Exercise Science Research Institute Ulster University Jordanstown Northern Ireland United Kingdom

**Keywords:** exercise, glucose, IL‐6, insulin, muscle oxygenation, myokines

## Abstract

Whole‐body vibration (WBV) is an exercise mimetic that elicits beneficial metabolic effects. This study aims to investigate the effects of WBV amplitude on metabolic, inflammatory, and muscle oxygenation responses. Forty women and men were assigned to a high (HI; *n* = 20, Age: 31 ± 6 y) or a low‐amplitude group (LO; *n* = 20, Age: 33 ± 6 y). Participants engaged in 10 cycles of WBV [1 cycle =1 min of vibration followed by 30 s of rest], while gastrocnemius muscle oxygen consumption (mVO_2_) was assessed using near‐infrared spectroscopy (NIRS). Blood samples were collected PRE, POST, 1H, 3Hs, and 24H post‐WBV and analyzed for insulin, glucose, and IL‐6. In the LO group, Homeostatic Model Assessment for Insulin Resistant (HOMA‐IR) at 3 h (0.7 ± 0.2) was significantly lower compared to PRE (1.1 ± 0.2; *p* = 0.018), POST (1.3 ± 0.3; *p* = 0.045), 1H (1.3 ± 0.3; *p* = 0.010), and 24H (1.4 ± 0.2; *p* < 0.001). In addition, at 24H, HOMA‐IR was significantly lower in the LO when compared to the HI group (LO: 1.4 ± 0.2 vs. HI: 2.2 ± 0.4; *p* = 0.030). mVO_2_ was higher (*p* = 0.003) in the LO (0.93 ± 0.29 ml/min/100 ml) when compared to the HI group (0.63 ± 0.28 ml/min/100 ml). IL‐6 at 3H (LO: 13.2 ± 2.7 vs. HI: 19.6 ± 4.0 pg·ml^−1^; *p* = 0.045) and 24H (LO: 4.2 ± 1.1 vs. HI: 12.5 ± 3.1 pg·ml^−1^; *p* = 0.016) was greater in the HI compared to the LO group. These findings indicate that low‐amplitude WBV provides greater metabolic benefits compared to high‐amplitude WBV.

## Graphical abstract

Whole‐body vibration (WBV) can elicit beneficial metabolic effects; however, whether or not the amplitude of vibration alters these effects has yet to be elucidated. Findings from the present investigation indicate that low‐amplitude WBV provides greater metabolic benefits compared to high‐amplitude WBV.

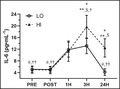

## 1 INTRODUCTION

Metabolic disorders, such as Type 2 diabetes (T2D) and metabolic syndrome, affect 35% of the United States population (Aguilar et al., 2015) and current trends indicate that two out of every five Americans will develop T2D in their lifetime (Prevention., C.f.D.C.a., & National Diabetes Statistics Report, 2017). It is estimated that only one in eight Americans is achieving optimal metabolic health (Araujo et al., 2019). Poor glucose metabolism, including tissue insulin resistance and reduced pancreatic beta‐cell function, precedes the development of hyperglycemia and subsequent T2D (Fonseca, 2009).

Physical inactivity increases the relative risk for developing T2D by up to 112% (Hamilton et al., 2014). Skeletal muscle is responsible for up to 90% of circulating glucose disposal (DeFronzo & Tripathy, 2009) and exercise‐induced enhancements in skeletal muscle VO_2_ have been shown to improve glycemic regulation in people with T2D (Russell et al., 2017). In addition, exercise increases skeletal muscle production of cytokines, known as myokines, which act as paracrine, endocrine, and autocrine signals (Pedersen et al., 2001). Myokines regulate a variety of factors, including muscle size (Lee & Jun, 2019) and macronutrient metabolism (Ahima & Park, 2015; Leal et al., 2018). Arguably, the most studied myokine is interleukin‐6 (IL‐6). Although high basal concentrations of IL‐6 are regarded to be inflammatory and have been linked to insulin resistance (Leal et al., 2018), acute exercise‐induced IL‐6 that is produced by skeletal muscle can increase both insulin sensitivity and glucose uptake (Glund et al., 2007). Despite the overall benefits provided by exercise, most individuals at risk for T2D cannot or choose not to adhere to regular exercise (Pietiläinen et al., 2008).

Whole‐body vibration (WBV) has emerged as an exercise mimetic that may be more tolerable than traditional modes of exercise, such as treadmill walking/running or cycling (Zago et al., 2018). Similar to traditional exercise modalities, WBV can elicit beneficial effects on metabolic health. In fact, a single bout of WBV increases circulating concentrations of IL‐6, which correspond with the normalization of glucose and insulin in obese individuals (Blanks et al., 2020). During bouts of traditional exercise, improvements in glucose metabolism are intensity‐dependent (Clark et al., 2003; Richter et al., 1985). A greater WBV amplitude appears to contribute to greater increases in muscle activation when compared to lower amplitude (Alizadeh‐Meghrazi et al., 2014), however, a greater WBV amplitude is accompanied by an increase in perceived exertion (Cochrane et al., 2008). Furthermore, this increased perceived exertion is thought to reduce exercise adherence (Witlox et al., 2019). WBV undoubtedly provides beneficial changes in glucose metabolism, however, it is unclear if a low WBV amplitude can induce similar metabolic responses as a high WBV amplitude. Accordingly, the aim of this study is to compare metabolic, myokine, and muscle oxygenation responses between High (HI) and Low (LO) amplitude WBV. It is hypothesized that LO will have greater metabolic, myokine, and muscle oxygenation responses when compared to the HI WBV.

## 2 METHODS

Forty healthy men and women ages 18–45 years old were recruited from a larger twin study to take part in this study. The 40 twins included 12 twin pairs and 16 singletons. Each twin set was paired together and assigned into the same amplitude group. Seven sets out of the 12 twin pairs were randomly assigned into low amplitude, while five sets were randomly assigned into high amplitude. Since the twin pairs are not independent participants, statistical analysis was used to take care of this non‐independence. Participants were excluded if they had a clinical diagnosis of cardiovascular disease, cancer, or chronic obstructive pulmonary disease. All study protocols were approved by the Institutional Review Board at Augusta University.

### 2.1 Experimental design

All participants reported to the Laboratory of Integrative Vascular and Exercise Physiology (LIVEP) at the Georgia Prevention Institute following an overnight fast and having abstained from moderate‐to‐vigorous physical activity for 24 h prior to investigation. The study visit consisted of the informed consent process, anthropometric measures, and body composition assessment. Twenty participants each were randomly assigned to either the HI or the LO group.

### 2.2 Participants’ characteristics, clinical laboratory values, and IL‐6

Height and weight were determined using a stadiometer and a standard platform scale (CN20, DETECTO^©^) and used for calculation of body mass index (BMI). Total body fat was determined using dual‐energy X‐ray absorptiometry (QDR‐4500W; Hologic). Resting systolic and diastolic blood pressures were evaluated using established protocols (Kapuku et al., 1999). An intravenous catheter was inserted into an antecubital vein and blood was separated via centrifugation and plasma samples were aliquoted, flash‐frozen in liquid nitrogen, and stored at −80°C until analysis. Blood samples were obtained at baseline (PRE), immediately after (POST), 1 h (1H), and 3 h (3H) post‐WBV. Twenty four hours (24H) following the acute WBV session, participants returned to the LIVEP in a fasted state and a venous blood sample was obtained. Fasting concentrations of total cholesterol (TC), high‐density lipoproteins (HDL), low‐density lipoproteins (LDL), triglycerides (TG), glucose, insulin, hemoglobin, and hematocrit were assessed using standard core laboratory techniques (Laboratory Corporation of America Holdings). In order to assess basal systemic inflammatory status, C‐reactive protein (CRP) within the detection range of 0.064–16.0 mg/L, was determined from PRE plasma samples using Simple Plex cartridges run on the Ella platform. The homeostasis model assessments‐2 for insulin resistance (HOMA‐IR) and beta‐cell function (HOMA β‐cell function) were calculated for all time points using circulating glucose and insulin concentrations (Matthews et al., 1985). Plasma concentrations of IL‐6, within the detection range of 0.26–16.3 pg/ml, were determined for each time point using microfluidic ELISA Simple Plex cartridges run on the Ella platform (ProteinSimple) according to the manufacturer's instructions.

### 2.3 Whole body vibration protocol

A synchronous whole‐body vibration platform (Power Plate Pro 5, Performance Health Systems) was used for this investigation. Participants were instructed to remove any footwear and to stand mid‐center on the platform with a loose grip on the front handrails. Participants were instructed to stand in a static squat position, consisting of knee flexion (~60°) with a stable non‐flexed trunk. Vibration frequency was set to 30 Hz as this frequency has been demonstrated to elicit the highest amount of gastrocnemius activation compared to other vibration frequencies (Cardinale et al., 2007), yet is well below the frequency in which potential harm may occur (Cardinale & Wakeling, 2005). The vibration amplitude was set to 5 and 2.5 mm for the HI and LO group, respectively. These settings yielded a calculated peak acceleration of 9.0 g (88.7 ms^−2^) for the HI group and 4.5 g (44.4 ms^−2^) for the LO group (Rauch et al., 2010), and intensity dials of 150 and 75 W/m^2^, respectively. The WBV protocol consisted of 10 cycles of 1 min of vibration exercise, followed by 30 s of standing rest, which has been demonstrated to induce changes in the metabolic and myokine profile (Blanks et al., 2020).

### 2.4 Muscle oxygenation

Oxygen consumption was measured during the WBV exercise using Near‐Infrared Spectroscopy (NIRS) as previously described (Colier et al., 1995). Prior to initiation of WBV, a continuous‐wave NIRS device (Portalite, Artinis Medical Systems) was attached to the muscle belly of the medial portion of the right gastrocnemius using tape. A cover was used to shield the probe from ambient light and minimize the change in contact pressure while allowing it to move with the skin during contractions. Emitted light wave‐lengths of 760 and 850 nm were used to detect relative changes in concentrations of oxygenated hemoglobin (O_2_Hb), deoxygenated hemoglobin (HHb), total hemoglobin (tHb = O_2_Hb + HHb), and Hemoglobin difference (Hbdiff = HHb – O_2_Hb). Light wavelengths were emitted from LEDs with an inter‐optode distance of 4 cm, thereby allowing for a theoretical penetration depth of 2 cm (Chance et al., 1992). A differential path‐length factor of four was used to correct for photon scattering within the tissue and data were collected at 10 Hz (Oxysoft; Artinis Medical Systems). Tissue saturation index (TSI), a measure of percent absolute oxygenated hemoglobin, was measured using multi‐distance algorithms incorporated into the Oxysoft software from three tissue depths. The thickness of adipose tissue superficial to the muscle belly of the gastrocnemius where the NIRS device was attached was determined using B‐mode ultrasound (Logiq 7, GE Medical Systems). After correcting for blood volume change (Ryan et al., 2012), an estimate of muscle oxygen consumption (mVO_2_) was obtained from the average of %Hbdiff signal during each minute of WBV and converted into milliliters of O_2_ per minute per 100 g of tissue {m $\dot{V}$ O_2_ [ml O_2_ min^−1^ (100 g) − 1] = abs[([HbDiff/2] × 60)/(10 × 1.04) × 4] × 22.4/1000}, assuming 22.4 L for the volume of gas (STPD) and 1.04 kg l−1 for muscle density [(Lucero et al., 2018; Sanni & McCully, 2019)].

### 2.5 Cardiopulmonary variables

Measures of cardiopulmonary variables were assessed to control for cardiorespiratory load between WBV amplitudes. During the WBV protocol, signal‐morphology impedance thoracic cardiography and high‐definition impedance (PhysioFlow^®^, Manatec Biomedical) were used to measure heart rate, stroke volume (SV), stroke volume indexed to body surface area (SVi), cardiac output (CO), cardiac output indexed to body surface area (CI), systemic vascular resistance (SVR), and systemic vascular resistance indexed to body surface area (SVRi). Oxygen saturation (SpO_2_) was obtained using an Onyx II fingertip sensor (Nonin Medical). Expired gases were collected breath‐by‐breath using a TruOne 2400 metabolic cart (Parvo Medics) and 30‐s averages were used to obtain oxygen consumption (VO_2_) during each cycle of WBV. Systemic oxygen extraction (O_2_Ex) was calculated using a derivative of the Fick equation (O_2_Ex =100 – [SpO_2_ − VO_2_/13.9 × CO × C]) (Jubran et al., 1998). All cardiopulmonary variables are reported as average values during each 1‐minute vibration session throughout the entire WBV protocol.

### 2.6 Statistical analyses

All statistical analyses were performed using SPSS version 25 (IBM Corporation). In order to control for the dependence of twin pairs, generalized estimating equations (GEE) were performed to identify group differences (i.e., LO vs. HI) in demographics and clinical laboratory markers. GEE is a multiple regression techniques that allows for non‐independence of twin or family data yielding unbiased standard errors and *p*‐values. Excess adiposity has been shown to significantly impact the metabolic and myokine responses to WBV (Blanks et al., 2020). Additionally, cholesterol has been shown to have a significant impact on muscle oxygenation (Leea, 2009). To account for these confounding effects, body fat and total cholesterol were analyzed as covariates for all WBV response variables. Multilevel linear mixed models were used to test for the group, time and group*time interaction effects on metabolic parameters and IL‐6 response to WBV protocol with families (i.e., twins) and individuals used as random effects. A significant overall time effect was followed by post estimation pairwise comparisons and multiple‐comparison adjustment was conducted using Bonferroni correction. Generalized estimating equations were also used to test for group differences in muscle oxygenation, tissue saturation, and cardiopulmonary variables. A priori sample size calculations based on previously published data (Blanks et al., 2020) determined that 18 participants per group would be needed to detect statistically significant differences in HOMA‐IR following WBV (*α* = 0.05 and power = 95%). Values are presented as mean ± SEM unless otherwise noted. An alpha <0.05 was considered statistically significant for all analyses.

## 3 RESULTS

### 3.1 Participant characteristics and laboratory values

Participant characteristics and clinical laboratory values are presented in Table 1. There was a significant difference in a race among the two groups, therefore race was used as a covariate in the analysis for all variables. Hemoglobin and Hematocrit were significantly greater in the LO group compared to the HI group. All other characteristics and laboratory values were similar between groups.

**TABLE 1:** Participant demographics & laboratory values

Variable	Low‐amplitude	High‐amplitude	*p*‐value
	(LO)	(HI)	
	*n* = 20	*n* = 20	
Sex (M/F)	6/14	5/15	0.78
Race (black/white)	3/17	13/7	**0.002** *
Age (y)	32.8 ± 1.7	30.7 ± 1.5	0.351
Height (cm)	165.0 ± 2.0	170.3 ± 2.0	0.062
Weight (kg)	79.7 ± 8.5	100.6 ± 7.9	0.073
Body mass index (kg·m^−2^)	30.1 ± 2.7	33.8 ± 2.6	0.323
Body fat (%)	40.2 ± 2.0	44.3 ± 1.9	0.129
Systolic blood pressure (mm Hg)	117.4 ± 2.8	119.3 ± 2.7	0.613
Diastolic blood pressure (mm Hg)	73.5 ± 1.8	73.6 ± 1.7	0.981
Clinical laboratory values			
Total cholesterol (mg·dl^−1^)	189 ± 10	170 ± 10	0.153
HDL (mg·dl^−1^)	54 ± 3	49 ± 3	0.255
LDL (mg·dl^−1^)	103 ± 10	100 ± 9	0.814
TC:HDL	3.9 ± 0.4	3.7 ± 0.4	0.743
VLDL	31 ± 4	20 ± 4	0.070
Triglycerides (mg·dl^−1^)	165 ± 27	102 ± 26	0.093
Hemoglobin (g·dl^−1^)	13.8 ± 0.4	12.6 ± 0.3	**0.014** *
Hematocrit (%)	41.5 ± 0.9	38.7 ± 0.9	**0.039** *
C‐reactive protein (mg·L^−1^)	0.57 ± 0.3	0.60 ± 0.3	0.951

Note

Generalized estimating equations. Mean and standard error.

Abbreviations: HDL, High‐density lipoprotein; LDL, Low‐density lipoprotein; TC HDL, Total cholesterol HDL ratio.

* *p* < 0.05 (bold).

### 3.2 Metabolic response to WBV

**FIGURE 1:**
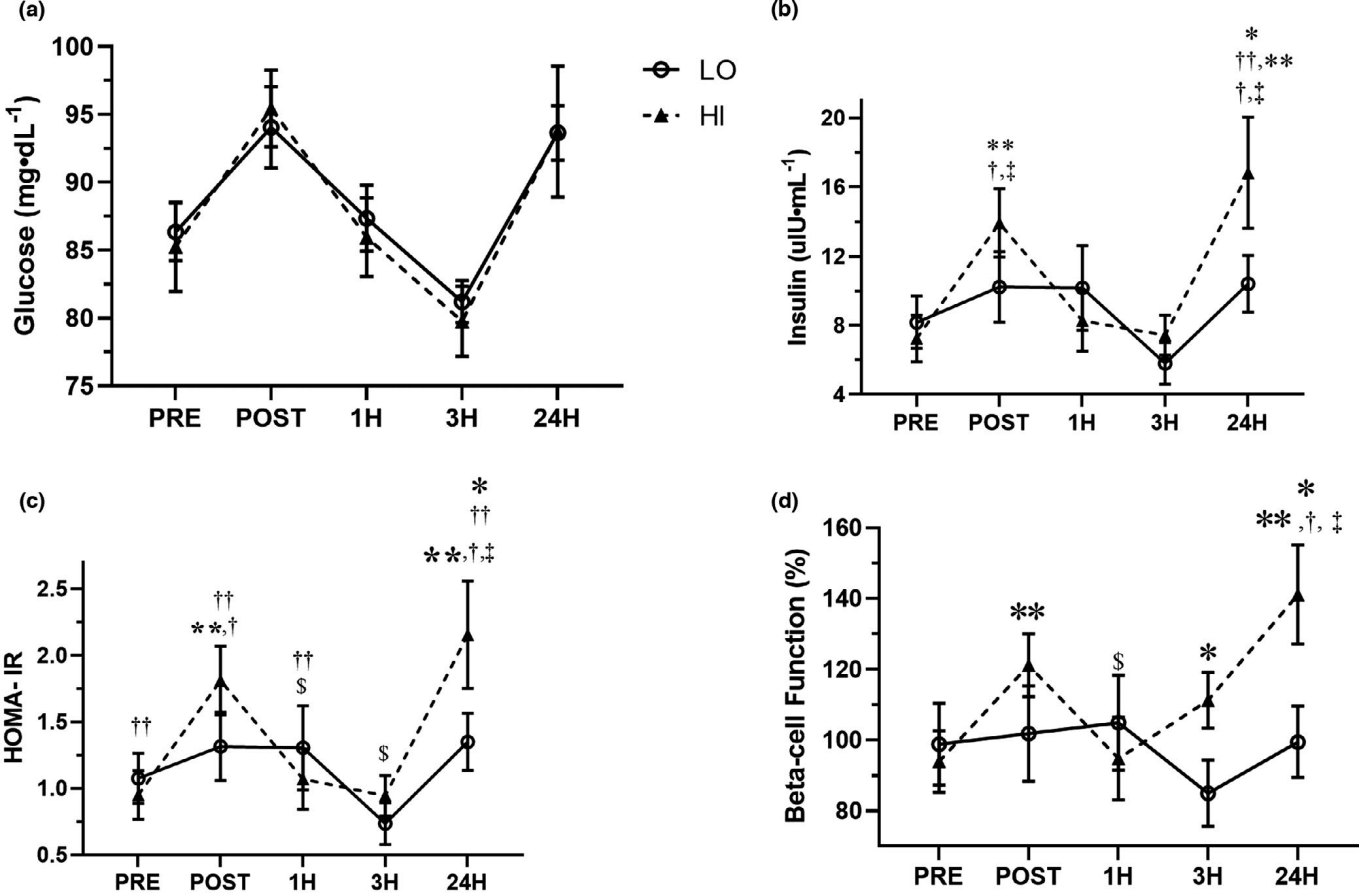
Time‐course of glucose (a), insulin (b), HOMA‐IR (c), and beta‐cell function (d) in response to low‐amplitude (LO‐circles) and high‐amplitude (HI‐triangles) whole body vibration. *p < 0.05 between groups, **p < 0.05 versus PRE in the HI group, ^$^ *p* < 0.05 versus POST in the HI group, ^†^ *p* < 0.05 versus 1H in the HI group, ^‡^versus 3H in the HI group, ^#^ *p* < 0.05 versus 1H in the LO group ^††^ *p* < 0.05 versus 3H in the LO group; Linear mixed model with repeated measures

Figure 1a illustrates the time‐course of the glucose response to WBV for both groups. There was no significant group by time interaction observed in blood glucose (*F* = 0.30, *p* = 0.88), however, there was a main effect of time (*F* = 28.08, *p* < 0.01). Cholesterol had a significant covariate effect (*F* = 7.37, *p* = 0.01) on glucose.

A significant group by time interaction (*F* = 2.88, *p* = 0.03) was observed for insulin (Figure 1b). Specifically, insulin in the HI group at POST (14.3 ± 2.1 µIU·ml^−1^; *p* < 0.001) and 24H (17.2 ± 2.1 µIU·ml^−1^; *p* = 0.004) was significantly greater compared to PRE (7.2 ± 3.2 µIU·ml^−1^), 1H (8.3 ± 1.8 µIU·ml^−1^; *p* = 0.001) and 3H (7.4 ± 1.2 µIU·ml^−1^; *p* < 0.001). Furthermore, insulin was significantly greater in the HI compare to LO (HI: 16.8 ± 3.2 µIU·ml^−1^; *p* = 0.031 vs. LO: 10.4 ± 1.6) at 24 h. Cholesterol had a significant covariate effect (*F* = 5.15, *p* = 0.03) on insulin.

A significant group by time interaction (*F* = 3.40, *p* = 0.011) was observed for HOMA‐IR (Figure 1c). In the HI group, HOMA‐IR was significantly higher in POST (1.8 ± 0.3; *p* < 0.001) and 24H (2.2 ± 0.4; *p* = 0.003) compared to PRE (1.0 ± 0.2) and 1H (1.1 ± 0.2), and was significantly higher at POST (1.8 ± 0.3; *p* < 0.001) and 24H (2.2 ± 0.4; *p* < 0.001) compared to 3H (0.9 ± 0.2). In the LO group, HOMA‐IR was significantly lower at 3H (0.7 ± 0.2) compared to PRE (1.1 ± 0.2; *p* = 0.018), POST (1.3 ± 0.3; *p* = 0.045), 1H (1.3 ± 0.3; *p* = 0.010), and 24H (1.4 ± 0.2; *p* < 0.001). Moreover, HOMA‐IR was significantly greater in the HI than the LO at 24H (HI: 2.2 ± 0.4; *p* = 0.030 vs. LO: 1.4 ± 0.2). Cholesterol had a significant covariate effect (*F* = 8.75, *p* < 0.01) on HOMA‐IR.

A significant group by time interaction (*F* = 3.70, *p* = 0.007) was observed for beta‐cell function (Figure 1d). Beta‐cell function was not significantly changed in the LO group over time. In the HI group, beta‐cell function was significantly greater at POST (121.1 ± 8.8%; *p* = 0.002) and 24H (141.1 ± 14.0%; *p* = 0.002) than PRE (94.0 ± 8.7). In addition, beta‐cell function was significantly greater at POST (121.1 ± 8.8%) compared to 1H (94.8 ± 11.7%; *p* = 0.042) and was significantly greater at 24H (141.1 ± 14.0) compared to 1H (94.8 ± 11.7; *p* = 0.009) and 3H (111.2 ± 7.9%; *p* = 0.021). Furthermore, beta‐cell function was significantly greater in the HI group compared to the LO group at 3H (HI: 111.2 ± 7.9% vs. LO: 85.0 ± 9.4; *p* = 0.019) and 24H (HI: 141.1 ± 14.0% vs. LO: 99.5 ± 10.1; *p* = 0.010).

### 3.3 Muscle oxygenation responses to WBV

**FIGURE 2:**
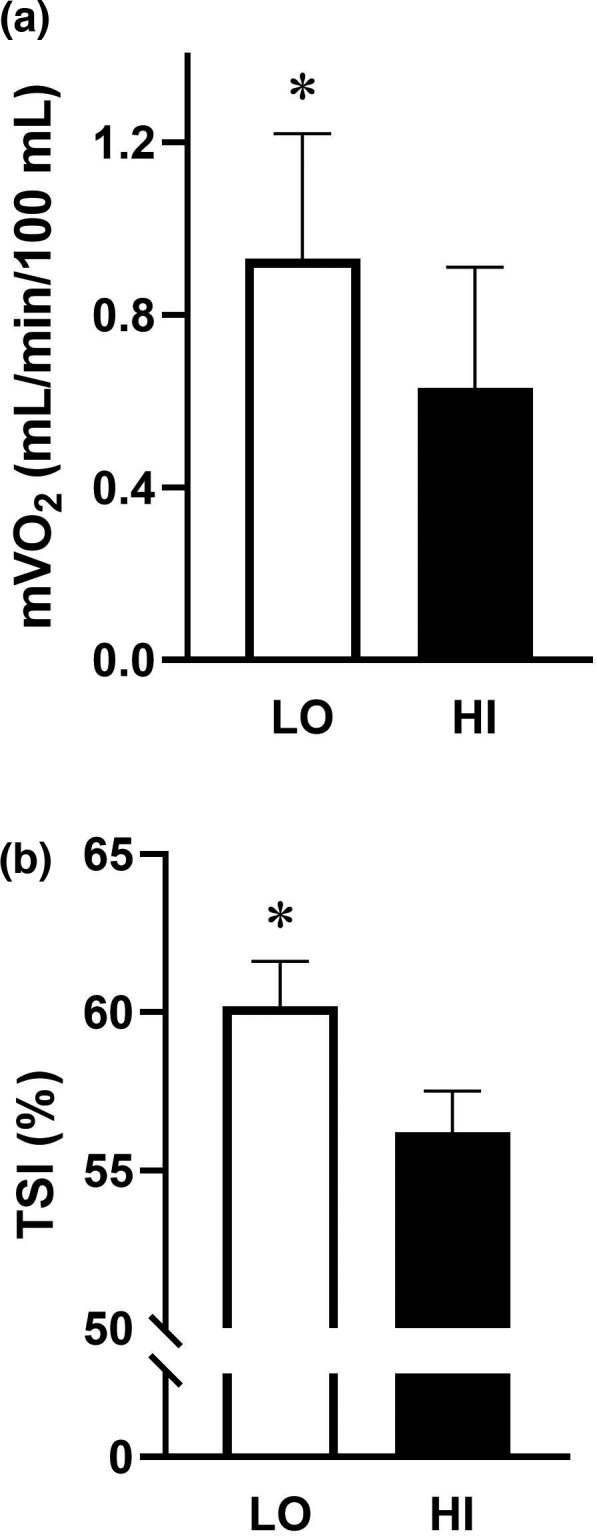
Average muscle oxygen consumption‐mVO_2_ (A) and tissue saturation index‐TSI (B) during low‐amplitude and high‐amplitude whole body vibration. *p < 0.05 between groups; Generalized estimating equations

Average muscle oxygen consumption (mVO_2_) to the gastrocnemius was significantly (*p* = 0.003) greater in the LO group (0.93 ± 0.29 ml/min/100 ml) when compared to the HI group (0.63 ± 0.28 ml/min/100 ml; Figure 2a). In addition, the average TSI was significantly (*p* = 0.037) greater in the LO group (60.2 ± 1.4%) compared to the HI group (56.2 ± 1.3%; Figure 2b). Average TSI was associated with HOMA‐IR at POST (*r* = −0.330, *p* = 0.023) and 3H (*r* = −0.317, *p* = 0.028).

### 3.4 Cardiopulmonary responses to WBV

The cardiopulmonary responses to WBV are presented in Tables 2 and 3. Average SV was significantly greater in the HI group than the LO group (*p* = 0.02). However, average SVi was not significantly different between groups (*p* = 0.13). In addition, CO (*p* = 0.06) and COi (*p* = 0.16) were not significantly different between groups. All other average cardiopulmonary variables were similar between groups throughout the WBV protocol.

**TABLE 2:** Baseline cardiopulmonary measures

Variable	LO	HI	*p*‐value
RER	0.29 ± 0.03	0.32 ± 0.03	0.393
Systemic oxygen extraction	0.31 ± 0.02	0.28 ± 0.02	0.459
Mean arterial pressure (mm Hg)	94.9 ± 2.5	103.9 ± 2.3	**0.011** *
SVR (dyn.s/cm^5^)	997.9 ± 41.6	1010.9 ± 41.9	0.828
SVRi (dyn.s/cm^5^·m^2^)	1929.8 ± 92.7	2066.6 ± 127.6	0.382
Heart rate (bpm)	86.6 ± 3.0	86.2 ± 2.1	0.907
Stroke volume (ml)	81.7 ± 2.9	91.6 ± 3.4	0.130
Stroke volume index (ml/m^2^)	43.9 ± 1.3	45.5 ± 1.4	0.443
Cardiac output (L/min)	7.4 ± 0.4	7.8 ± 0.3	0.362
Cardiac output index (L/min/m^2^)	3.8 ± 0.2	3.9 ± 0.2	0.684

Note

Average of a cardiopulmonary base to whole‐body vibration in low‐amplitude (LO) and high‐amplitude (HI) groups. Mean and standard error.

Abbreviations: RER, respiratory exchange ratio; SVR, systemic vascular resistance; SVRi, systemic vascular resistance index (SVRi); VO_2_, oxygen consumption.

* *p* < 0.05 between groups (bold); Generalized estimating equations.

**TABLE 3:** Cardiopulmonary responses

Variable	LO	HI	*p*‐value
VO_2_ (L/min)	0.72 ± 0.07	0.79 ± 0.08	0.502
VO_2_ (ml/kg/min)	9.0 ± 0.6	8.69 ± 0.5	0.684
RER	1.01 ± 0.02	1.03 ± 0.02	0.679
Systemic oxygen extraction	0.31 ± 0.02	0.29 ± 0.02	0.552
Mean arterial pressure (mm Hg)	99.5 ± 3.9	104.8 ± 4.0	0.427
SVR (dyn.s/cm^5^)	613.9 ± 29.2	569.1 ± 31.1	0.293
SVRi (dyn.s/cm^5^·m^2^)	1186.1 ± 74.4	1173.7 ± 77.1	0.909
Heart rate (bpm)	134.0 ± 4.7	135.7 ± 4.9	0.797
Stroke volume (ml)	94.4 ± 4.9	110.3 ± 5.0	**0.024** *
Stroke volume index (ml/m^2^)	49.1 ± 1.5	52.3 ± 1.5	0.128
Cardiac output (L/min)	12.7 ± 0.8	14.9 ± 0.8	0.064
Cardiac output index (L/min/m^2^)	6.6 ± 0.3	7.1 ± 0.4	0.275

Note

Average cardiopulmonary responses to whole‐body vibration in low‐amplitude (LO) and high‐amplitude (HI) groups. Mean and standard error.

Abbreviations: RER, respiratory exchange ratio; SVR, systemic vascular resistance; SVRi, systemic vascular resistance index (SVRi); VO_2_, oxygen consumption.

* *p* < 0.05 between groups (bold); Generalized estimating equations.

### 3.5 Myokine response to WBV

**FIGURE 3:**
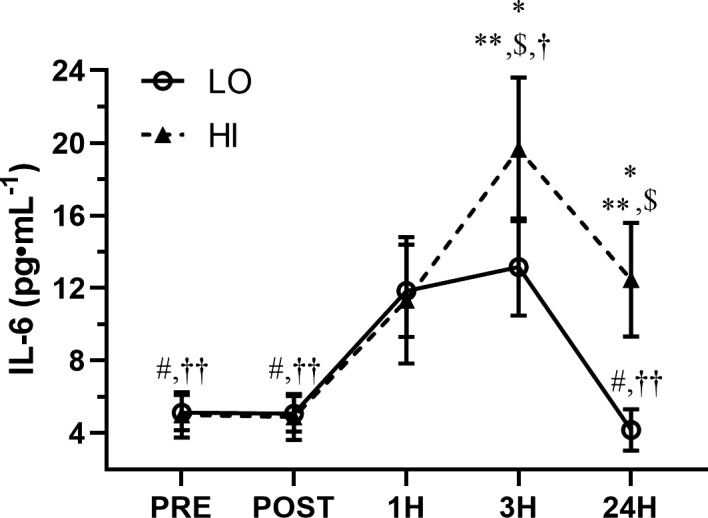
Time‐course of IL‐6 in response to low‐amplitude (LO) and high‐amplitude (HI) whole body vibration. *p < 0.05 between groups, **p < 0.05 versus PRE in the HI group, ^$^ *p* < 0.05 versus POST in the HI group, ^†^ *p* < 0.05 versus 1H in the HI group, ^††^ *p* < 0.05 versus 3H in the HI group, ^#^ *p* < 0.05 versus 1H in the LO group, ^††^ *p* < 0.05 versus 3H in the LO group. Linear mixed model with repeated measures

Figure 3 illustrates the time‐course of the IL‐6 response to WBV for both groups. There was no significant amplitude × time interaction (*F* = 2.22, *p* = 0.07); however, there was a significant overall main effect for concentrations of IL‐6 for both amplitude (*F* = 4.22, *p* = 0.47) and time (*F* = 11.76, *p* < 0.01). Bodyfat has a significant covariate effect (*F* = 13.2, *p* < 0.01) on IL‐6.

## 4 DISCUSSION

Acute whole‐body vibration alters glucose metabolism and myokine responses, both of which may reduce the risk of developing metabolic diseases such as Type 2 diabetes and metabolic syndrome. Whether or not increasing the amplitude of WBV offers greater physiological effects has yet to be elucidated. Accordingly, the present investigation sought to determine the effect of WBV amplitude on the metabolic, muscle oxygenation, and myokine responses. Findings demonstrate that both high‐amplitude and low‐amplitude WBV elicit metabolic and myokine responses in a sample of the general population. In addition, we present data for the first time to demonstrate that compared to high‐amplitude WBV, low‐amplitude WBV elicits greater muscle oxygenation and this response coincided with more favorable metabolic responses.

### 4.1 Metabolic response to WBV

Traditional forms of exercise, such as treadmill walking/running or cycling, have been shown to alter glucose metabolism in an exercise intensity dependent manner (Haddad et al., 2003). However, lower intensity exercise increases exercise adherence in comparison to higher intensity exercise of the same frequency and duration (Tse et al., 2015). Recently, WBV has emerged as an exercise mimetic that is capable of inducing alterations in glucose metabolism similar to those that occur in response to traditional exercise (Blanks et al., 2020; Gomes‐Neto et al., 2019). Consistent with previous investigations (Blanks et al., 2020; Gomes‐Neto et al., 2019), the current study demonstrated a significant reduction in blood glucose in response to acute WBV that was independent of the exercising amplitude. In the LO group, a significant reduction in insulin resistance was observed 3 h post‐WBV which could result in an increase in glucose metabolism. This result is consistent with our previous report of significant metabolic alterations occurring 3 h following an acute bout of WBV (Blanks et al., 2020). In addition, a single session of WBV has been shown to improve circulating glucose in women with type 2 diabetes (Pessoa et al., 2018) and 8 weeks of WBV can improve glucose concentrations to a similar degree as aerobic exercise in males with diabetes (Behboudi et al., 2011). Traditional exercise increases skeletal muscle glucose uptake by increasing myocyte sensitivity to insulin as well as by increasing GLUT4 glucose transport, independent of insulin (Richter & Hargreaves, 2013). Although not measured in the current investigation, WBV likely induced similar alterations in GLUT4 transport, thereby reducing insulin resistance in the LO group. However, compared to the LO group, a post‐WBV reduction in HOMA‐IR was not observed in the HI group due to differential insulin responses. During bouts of acute exercise, pancreatic insulin secretion increases in an intensity‐dependent manner (Malin et al., 2016). Although speculative, this mechanism likely explains the significant increases in insulin and HOMA‐IR observed in the HI group, despite the post‐WBV glucose response that was similar to the LO group. Moreover, high‐amplitude WBV elicited an improvement in beta‐cell function which led to a significant difference in beta‐cell function between groups at 3H and 24H. Although high‐amplitude WBV appears to have a beneficial impact on beta‐cell function, this may be an attempt to compensate for the lack of improvement in insulin resistance. Taken together, these results suggest that although both high and low‐amplitude WBV provides favorable metabolic benefits, low‐amplitude WBV provides a more robust HOMA‐IR response in comparison to high‐amplitude WBV.

### 4.2 Muscle oxygenation response to WBV

An increase in mVO_2_ contributes to the improvement in glucose metabolism (Baron & Clark, 1997; Kemppainen et al., 2003). During exercise, skeletal muscle oxygenation is increased in an intensity‐dependent manner to meet metabolic demand for oxygen and nutrients (Joyner & Casey, 2015). However, intense isometric exercise, such as the static squat that was performed by participants in the current investigation, has been shown to limit mVO_2_ in the working muscles (McNeil et al., 2015). The findings of the present study are consistent with previous observations that demonstrated a reduced mVO_2_ in the working muscle during a higher intensity WBV exercise. Accordingly, it is plausible that the HI group experienced more intense muscle contractions that led to reduced blood flow to the gastrocnemius thereby leading to reduced mVO_2_ in the gastrocnemius muscle when compared to the LO group. In addition, gravitational forces generated during WBV have been shown to increase activation of the gastrocnemius, vastus lateralis, and vastus medialis in an intensity‐dependent manner (Roelants et al., 2006). Thus, it cannot be ruled out that high‐amplitude WBV could elicit greater increases in mVO_2_ to the vastus lateralis and vastus medialis, thereby resulting in lower mVO_2_ to the gastrocnemius in the HI group compared to the LO group. Future studies are warranted to evaluate the distribution of muscle mVO_2_ during WBV. Nonetheless, low tissue oxygenation plays an important role in the pathogenesis and progression of T2D as glucose concentration sensing is dependent on oxygenation (Wilson & Matschinsky, 2019). In addition, data support that the rate of muscle oxygen saturation is dependent on WBV type (Games et al., 2015). In addition to the evidence of higher mVO_2_ in the LO group, there was a significant inverse relationship between tissue saturation index and HOMA‐IR at different time points. Taken together, these data suggest that low‐amplitude WBV elicits greater increases in gastrocnemius muscle oxygenation, which appears to contribute to more favorable improvements in glucose metabolism compared to high‐amplitude WBV.

### 4.3 Myokine response to WBV

Myokines are produced by contracting skeletal muscle and have a variety of autocrine, paracrine, and endocrine functions. IL‐6 is the most readily produced myokine by skeletal muscle (Leal et al., 2018). Although chronically elevated basal concentrations of IL‐6 have been linked to both inflammation and disease, transient elevations in IL‐6 produced by skeletal muscle act as an “energy sensor” and enhance glucose metabolism (Keller et al., 2001). Recently, our lab has demonstrated that a single bout of WBV can transiently increase circulating IL‐6 (Blanks et al., 2020). The findings of the present investigation are consistent with previous data and provide evidence that the 3 h increase in circulating IL‐6 and the concominant decrease in glucose is independent of WBV amplitude. In addition, the peak IL‐6 response in the current study not only occurred 3 h following completion of the WBV protocol, but the peak also corresponded with the most favorable metabolic improvements in both groups. Muscle activation during exercise elicits increases in IL‐6 release from skeletal muscle in an intensity‐dependent manner (Pedersen et al., 2001). Although an increase in IL‐6 concentration has been observed after an acute eccentric exercise, concentric exercise did not elicit an IL‐6 response (Bruunsgaard et al., 1997; Hellsten et al., 1997; Koenig et al., 2016) and suggest that the increase in IL‐6 might be related to microtear of the muscle. In contrast, 4 h of simulated multi‐axial vibration did not increase creatine kinase, albeit an increase in circulating concentrations of IL‐6 (Kia et al., 2020). Although muscle activation was not assessed in the current investigation, muscle activation is increased with increasing vibration amplitude (Alizadeh‐Meghrazi et al., 2014). Accordingly, the lower peak IL‐6 response observed in the LO group is likely due to less muscle activation compared to what the HI group experienced. Moreover, numerous investigations have demonstrated significant IL‐6 production by skeletal muscle in response to the contraction in a time‐course similar to that of the current investigation (Garneau et al., 2020; Keller et al., 2001; Schild et al., 2016). Although the source of IL‐6 cannot be confirmed by the present investigation, it is very likely that post‐WBV increases in IL‐6 were derived from skeletal muscle; however additional sources of IL‐6 cannot be ruled out. Nonetheless, peak concentrations of WBV‐induced IL‐6 corresponded with the most favorable alterations in glucose metabolism in both groups. Taken together, these data suggest that both low and high‐workload acute WBV induce beneficial increases in circulating concentrations of IL‐6.

### 4.4 Experimental considerations

Although the present investigation utilized a cross‐sectional design without a no vibration control, we believe that this approach most effective design for this first of its kind study. In addition, both amplitude groups were instructed to stand on the vibration platform in the same squat position to help control for the effects of a static squat. It is also important to note that differences in hemoglobin and hematocrit were observed between the two groups and could have impacted the NIRS data. However, it is unlikely that the slight differences observed would affect the rate of oxygen delivery and subsequent muscle oxygen consumption. Nonetheless, future studies are needed to investigate the effect of hemoglobin and hematocrit on muscle oxygen consumption using NIRS.

## 5 CONCLUSION

For the first time, data from the present investigation demonstrate that acute synchronous whole‐body vibration elicits alterations in glucose metabolism and myokine production in the general population. Perhaps even more importantly, findings indicate that low‐amplitude WBV contributes to more favorable improvements in glucose metabolism and muscle oxygenation when compared to high‐amplitude WBV. Taken together, these results suggest that low‐workload WBV can elicit metabolic and skeletal muscle improvements and may represent a novel method to reduce the risk of metabolic diseases by improving muscle oxygen consumption and glucose metabolism. WBV represents a novel approach when prescribing exercise for those who show poor compliance or are unable or unwilling to use traditional modes of exercise.

## CONFLICT OF INTEREST

The authors declare no conflicts of interest.

## AUTHORS CONTRIBUTION

Study conceptualization and design; AMB, XW, RH. Acquisition, Analysis, and interpretation of data work; AAS, CCD, CH, RHC, JL, SS, KN, NY, JT, XW, RH. Manuscript draft and conceptual revision; AAS, AMB, CCD, XW, RH. Final approval of the manuscript; AAS, AMB, CCD, CH, RHC, JL, SS, KN, NY, JT, XW, RH.

## References

[phy215208-bib-0001] Aguilar, M. , Bhuket, T. , Torres, S. , Liu, B. , & Wong, R. J. (2015). Prevalence of the metabolic syndrome in the United States, 2003‐2012. JAMA, 313(19), 1973. 10.1001/jama.2015.4260 25988468

[phy215208-bib-0002] Ahima, R. S. , & Park, H. K. (2015). Connecting myokines and metabolism. Endocrinology and Metabolism, 30(3), 235. 10.3803/EnM.2015.30.3.235 26248861 PMC4595346

[phy215208-bib-0003] Alizadeh‐Meghrazi, M ., Masani, K ., Zariffa, J ., Sayenko, D. G. , Popovic, M. R. , & Craven, B. C . (2014). Effect of whole‐body vibration on lower‐limb EMG activity in subjects with and without spinal cord injury. The Journal of Spinal Cord Medicine, 37(5), 525–536. 10.1179/2045772314Y.0000000242 24986541 PMC4166187

[phy215208-bib-0004] Araujo, J. , Cai, J. , & Stevens, J. (2019). Prevalence of optimal metabolic health in American adults: National Health and Nutrition Examination Survey 2009–2016. Metabolic Syndrome and Related Disorders, 17(1), 46–52. 10.1089/met.2018.0105 30484738

[phy215208-bib-0005] Baron, A. D. , & Clark, M. G. (1997). Role of blood flow in the regulation of muscle glucose uptake. Annual Review of Nutrition, 17, 487–499. 10.1146/annurev.nutr.17.1.487 9240937

[phy215208-bib-0006] Behboudi, L. , Azarbayjani, M.‐A. , Aghaalinejad, H. , & Salavati, M. (2011). Effects of aerobic exercise and whole body vibration on glycaemia control in type 2 diabetic males. Asian Journal of Sports Medicine, 2(2), 83–90. 10.5812/asjsm.34789 22375223 PMC3289205

[phy215208-bib-0007] Blanks, A. M. , Rodriguez‐Miguelez, P. , Looney, J. , Tucker, M. A. , Jeong, J. , Thomas, J. , Blackburn, M. , Stepp, D. W. , Weintraub, N. J. , & Harris, R. A. (2020). Whole body vibration elicits differential immune and metabolic responses in obese and normal weight individuals. Brain, Behavior, & Immunity ‐ Health, 1, 100011. 10.1016/j.bbih.2019.100011 PMC8474538 38377415

[phy215208-bib-0008] Bruunsgaard, H. , Galbo, H. , Halkjaer‐Kristensen, J. , Johansen, T. L. , MacLean, D. A. , & Pedersen, B. K. (1997). Exercise‐induced increase in serum interleukin‐6 in humans is related to muscle damage. Journal of Physiology, 499 (Pt 3), 833–841. 10.1113/jphysiol.1997.sp021972 PMC1159298 9130176

[phy215208-bib-0009] Cardinale, M. , Ferrari, M. , & Quaresima, V. (2007). Gastrocnemius medialis and vastus lateralis oxygenation during whole‐body vibration exercise. Medicine and Science in Sports and Exercise, 39(4), 694–700. 10.1249/mss.0b013e31803084d8 17414808

[phy215208-bib-0010] Cardinale, M. , & Wakeling, J. (2005). Whole body vibration exercise: Are vibrations good for you? British Journal of Sports Medicine, 39(9), 585–589; discussion 589. 16118292 10.1136/bjsm.2005.016857 PMC1725325

[phy215208-bib-0011] Chance, B. , Dait, M. T. , Zhang, C. , Hamaoka, T. , & Hagerman, F. (1992). Recovery from exercise‐induced desaturation in the quadriceps muscles of elite competitive rowers. American Journal of Physiology‐Cell Physiology, 262(3), C766–C775. 10.1152/ajpcell.1992.262.3.C766 1312785

[phy215208-bib-0012] Clark, M. G. , Wallis, M. G. , Barrett, E. J. , Vincent, M. A. , Richards, S. M. , Clerk, L. H. , & Rattigan, S . (2003). Blood flow and muscle metabolism: A focus on insulin action. American Journal of Physiology‐Endocrinology and Metabolism, 284(2), E241–E258. 10.1152/ajpendo.00408.2002 12531739

[phy215208-bib-0013] Cochrane, D. J. , Sartor, F. , Winwood, K. , Stannard, S. R. , Narici, M. V. , & Rittweger, J . (2008). A comparison of the physiologic effects of acute whole‐body vibration exercise in young and older people. Archives of Physical Medicine and Rehabilitation, 89(5), 815–821. 10.1016/j.apmr.2007.09.055 18452726

[phy215208-bib-0014] Colier, W. N. J. M. , Meeuwsen, I. B. A. E. , Degens, H. , & Oeseburg, B. (1995). Determination of oxygen consumption in muscle during exercise using near infrared spectroscopy. Acta Anaesthesiologica Scandinavica, 107, 151–155. 10.1111/j.1399-6576.1995.tb04350.x 8599269

[phy215208-bib-0015] DeFronzo, R. A. , & Tripathy, D. (2009). Skeletal muscle insulin resistance is the primary defect in type 2 diabetes. Diabetes Care, 32(Suppl 2), S157–S163. 10.2337/dc09-S302 19875544 PMC2811436

[phy215208-bib-0016] Fonseca, V. A. (2009). Defining and characterizing the progression of type 2 diabetes. Diabetes Care, 32(Suppl 2), S151–S156. 10.2337/dc09-S301 19875543 PMC2811457

[phy215208-bib-0017] Games, K. E. , Sefton, J. M. , & Wilson, A. E. (2015). Whole‐body vibration and blood flow and muscle oxygenation: A meta‐analysis. Journal of Athletic Training, 50(5), 542–549. 10.4085/1062-6050-50.2.09 25974682 PMC4560014

[phy215208-bib-0018] Garneau, L. , Parsons, S. A. , Smith, S. R. , Mulvihill, E. E. , Sparks, L. M. , & Aguer, C. (2020). Plasma myokine concentrations after acute exercise in non‐obese and obese sedentary women. Frontiers in Physiology, 11, 18. 32132925 10.3389/fphys.2020.00018 PMC7040180

[phy215208-bib-0019] Glund, S. , Deshmukh, A. , Long, Y. C. , Moller, T. , Koistinen, H. A. , Caidahl, K. , Zierath, J. R. , & Krook, A . (2007). Interleukin‐6 directly increases glucose metabolism in resting human skeletal muscle. Diabetes, 56(6), 1630–1637. 10.2337/db06-1733 17363741

[phy215208-bib-0020] Gomes‐Neto, M. , de Sá‐Caputo, D. D. , Paineiras‐Domingos, L. L. , Brandão, A. A. , Neves, M. F. , Marin, P. J. , Sañudo, B. , & Bernardo‐Filho, M . (2019). Effects of whole‐body vibration in older adult patients with type 2 diabetes mellitus: A systematic review and meta‐analysis. Canadian Journal of Diabetes, 43(7), 524–529.e2. 10.1016/j.jcjd.2019.03.008 31104903

[phy215208-bib-0021] Haddad, E. , Wells, G. A. , Sigal, R. J. , Boulé, N. G. , & Kenny, G. P . (2003). Meta‐analysis of the effect of structured exercise training on cardiorespiratory fitness in type 2 diabetes mellitus. Diabetologia, 46(8), 1071–1081. 10.1007/s00125-003-1160-2 12856082

[phy215208-bib-0022] Hamilton, M. T. , Hamilton, D. G. , & Zderic, T. W. (2014). Sedentary behavior as a mediator of type 2 diabetes. Medicine and Sport Science, 60, 11–26. 25226797 10.1159/000357332 PMC4364419

[phy215208-bib-0023] Hellsten, Y. , Frandsen, U. , Orthenblad, N. , Sjødin, B. , & Richter, E. A. (1997). Xanthine oxidase in human skeletal muscle following eccentric exercise: A role in inflammation. Journal of Physiology, 498(Pt 1), 239–248. 10.1113/jphysiol.1997.sp021855 PMC1159248 9023782

[phy215208-bib-0024] Joyner, M. J. , & Casey, D. P. (2015). Regulation of increased blood flow (hyperemia) to muscles during exercise: A hierarchy of competing physiological needs. Physiological Reviews, 95(2), 549–601. 10.1152/physrev.00035.2013 25834232 PMC4551211

[phy215208-bib-0025] Jubran, A. , Mathru, M. , Dries, D. , & Tobin, M. J. (1998). Continuous recordings of mixed venous oxygen saturation during weaning from mechanical ventilation and the ramifications thereof. American Journal of Respiratory and Critical Care Medicine, 158(6), 1763–1769. 10.1164/ajrccm.158.6.9804056 9847265

[phy215208-bib-0026] Kapuku, G. K. , Treiber, F. A. , Davis, H. C. , Harshfield, G. A. , Cook, B. B. , & Mensah, G. A . (1999). Hemodynamic function at rest, during acute stress, and in the field: Predictors of cardiac structure and function 2 years later in youth. Hypertension, 34(5), 1026–1031. 10.1161/01.HYP.34.5.1026 10567177

[phy215208-bib-0027] Keller, C. , Steensberg, A. , Pilegaard, H. , Osada, T. , Saltin, B. , Pedersen, B. K. , & Neufer, P. D. (2001). Transcriptional activation of the IL‐6 gene in human contracting skeletal muscle: Influence of muscle glycogen content. The FASEB Journal, 15(14), 2748–2750. 10.1096/fj.01-0507fje 11687509

[phy215208-bib-0028] Kemppainen, J. , Stolen, K. , Kalliokoski, K. K. , Salo, T. , Karanko, H. , Viljanen, T. , Airaksinen, J. , Nuutila, P. , & Knuuti, J . (2003). Exercise training improves insulin stimulated skeletal muscle glucose uptake independent of changes in perfusion in patients with dilated cardiomyopathy. Journal of Cardiac Failure, 9(4), 286–295. 10.1054/jcaf.2003.35 13680549

[phy215208-bib-0029] Kia, K ., Fitch, S. M. , Newsom, S. A. , & Kim, J. H. (2020). Effect of whole‐body vibration exposures on physiological stresses: Mining heavy equipment applications. Applied Ergonomics, 85, 103065. 10.1016/j.apergo.2020.103065 32174353 PMC8117724

[phy215208-bib-0030] Koenig, R. T. , Dickman, J. R. , Kang, C.‐H. , Zhang, T. , Chu, Y.‐F. , & Ji, L. L. (2016). Avenanthramide supplementation attenuates eccentric exercise‐inflicted blood inflammatory markers in women. European Journal of Applied Physiology, 116(1), 67–76. 10.1007/s00421-015-3244-3 26289619

[phy215208-bib-0031] Leal, L. G. , Lopes, M. A. , & Batista, M. L. Jr . (2018). Physical exercise‐induced myokines and muscle‐adipose tissue crosstalk: A review of current knowledge and the implications for health and metabolic diseases. Frontiers in Physiology, 9, 1307. 30319436 10.3389/fphys.2018.01307 PMC6166321

[phy215208-bib-0032] Lee, J. H. , & Jun, H. S. (2019). Role of myokines in regulating skeletal muscle mass and function. Frontiers in Physiology, 10, 42. 30761018 10.3389/fphys.2019.00042 PMC6363662

[phy215208-bib-0033] Leea, V. C . (2009). Cholesterol and skeletal muscle health. World Review of Nutrition and Dietetics, 100, 71–79. 19696529 10.1159/000235713

[phy215208-bib-0034] Lucero, A. A. , Addae, G. , Lawrence, W. , Neway, B. , Credeur, D. P. , Faulkner, J. , Rowlands, D. , & Stoner, L . (2018). Reliability of muscle blood flow and oxygen consumption response from exercise using near‐infrared spectroscopy. Experimental Physiology, 103(1), 90–100. 10.1113/EP086537 29034529 PMC12884174

[phy215208-bib-0035] Malin, S. K. , Rynders, C. A. , Weltman, J. Y. , Barrett, E. J. , & Weltman, A. (2016). Exercise intensity modulates glucose‐stimulated insulin secretion when adjusted for adipose, liver and skeletal muscle insulin resistance. PLoS One, 11(4), e0154063. 10.1371/journal.pone.0154063 27111219 PMC4844153

[phy215208-bib-0036] Matthews, D. R. , Hosker, J. P. , Rudenski, A. S. , Naylor, B. A. , Treacher, D. F. , & Turner, R. C . (1985). Homeostasis model assessment: Insulin resistance and beta‐cell function from fasting plasma glucose and insulin concentrations in man. Diabetologia, 28(7), 412–419. 3899825 10.1007/BF00280883

[phy215208-bib-0037] McNeil, C. J. , Allen, M. D. , Olympico, E. , Shoemaker, J. K. , & Rice, C. L. (2015). Blood flow and muscle oxygenation during low, moderate, and maximal sustained isometric contractions. American Journal of Physiology: Regulatory, Integrative and Comparative Physiology, 309(5), R475–R481. 10.1152/ajpregu.00387.2014 PMC4591373 26084698

[phy215208-bib-0038] Pedersen, B. K. , Steensberg, A. , & Schjerling, P. (2001). Muscle‐derived interleukin‐6: Possible biological effects. Journal of Physiology, 536(Pt 2), 329–337. 10.1111/j.1469-7793.2001.0329c.xd PMC2278876 11600669

[phy215208-bib-0039] Pessoa, M. F. , Souza, H. C. , Silva, A. P. , Clemente, R. D. , & Brandão, D. C . (2018). Acute whole body vibration decreases the glucose levels in elderly diabetic women. Rehabilitation Research and Practice, 2018, 3820615. 29971166 10.1155/2018/3820615 PMC6008658

[phy215208-bib-0040] Pietiläinen, K. H. , Kaprio, J. , Borg, P. , Plasqui, G. , Yki‐Järvinen, H. , Kujala, U. M. , Rose, R. J. , Westerterp, K. R. , & Rissanen, A . (2008). Physical inactivity and obesity: A vicious circle. Obesity, 16(2), 409–414. 10.1038/oby.2007.72 18239652 PMC2249563

[phy215208-bib-0041] Prevention., C.f.D.C.a., National Diabetes Statistics Report . (2017). Centers for Disease Control and Prevention, US Department of Health and Human Services, 2017.

[phy215208-bib-0042] Rauch, F. , Sievanen, H. , Boonen, S. , Cardinale, M. , Degens, H. , Felsenberg, D. , Roth, J. , Schoenau, E. , Verschueren, S. , & Rittweger, J. (2010). Reporting whole‐body vibration intervention studies: Recommendations of the International Society of Musculoskeletal and Neuronal Interactions. Journal of Musculoskeletal and Neuronal Interactions, 10(3), 193–198. 20811143

[phy215208-bib-0043] Richter, E. A. , & Hargreaves, M. (2013). Exercise, GLUT4, and skeletal muscle glucose uptake. Physiological Reviews, 93(3), 993–1017. 10.1152/physrev.00038.2012 23899560

[phy215208-bib-0044] Richter, E. A. , Ploug, T. , & Galbo, H. (1985). Increased muscle glucose uptake after exercise: No need for insulin during exercise. Diabetes, 34(10), 1041–1048. 10.2337/diab.34.10.1041 3899806

[phy215208-bib-0045] Roelants, M. , Verschueren, S. M. , Delecluse, C. , Levin, O. , & Stijnen, V. (2006). Whole‐body‐vibration‐induced increase in leg muscle activity during different squat exercises. Journal of Strength and Conditioning Research, 20(1), 124–129. 16503671 10.1519/R-16674.1

[phy215208-bib-0046] Russell, R. D. , Hu, D. , Greenaway, T. , Blackwood, S. J. , Dwyer, R. M. , Sharman, J. E. , Jones, G. , Squibb, K. A. , Brown, A. A. , Otahal, P. , Boman, M. , Al‐Aubaidy, H. , Premilovac, D. , Roberts, C. K. , Hitchins, S. , Richards, S. M. , Rattigan, S. & Keske, M. A . (2017). Skeletal muscle microvascular‐linked improvements in glycemic control from resistance training in individuals with type 2 diabetes. Diabetes Care, 40(9), 1256–1263. 10.2337/dc16-2750 28687542

[phy215208-bib-0047] Ryan, T. E. , Erickson, M. L. , Brizendine, J. T. , Young, H.‐J. , & McCully, K. K . (2012). Noninvasive evaluation of skeletal muscle mitochondrial capacity with near‐infrared spectroscopy: Correcting for blood volume changes. Journal of Applied Physiology, 113(2), 175–183. 10.1152/japplphysiol.00319.2012 22582211 PMC3404707

[phy215208-bib-0048] Sanni, A. A. , & McCully, K. K. (2019). Interpretation of Near‐Infrared Spectroscopy (NIRS) signals in skeletal muscle. Journal of Functional Morphology and Kinesiology, 4(2), 28. 10.3390/jfmk4020028 PMC7739319 33467344

[phy215208-bib-0049] Schild, M. , Eichner, G. , Beiter, T. , Zügel, M. , Krumholz‐Wagner, I. , Hudemann, J. , Pilat, C. , Krüger, K. , Niess, A. M. , Steinacker, J. M. , & Mooren, F. C. (2016). Effects of acute endurance exercise on plasma protein profiles of endurance‐trained and untrained individuals over time. Mediators of Inflammation, 2016, 4851935. 27239103 10.1155/2016/4851935 PMC4867072

[phy215208-bib-0050] Tse, A. C. , Wong, T. W. , & Lee, P. H. (2015). Effect of low‐intensity exercise on physical and cognitive health in older adults: A systematic review. Sports Medicine—Open, 1(1), 37. 10.1186/s40798-015-0034-8 26512340 PMC4612316

[phy215208-bib-0051] Wilson, D. F. , & Matschinsky, F. M . (2019). Oxygen dependence of glucose sensing: Role in glucose homeostasis and related pathology. Journal of Applied Physiology, 126(6), 1746–1755. 10.1152/japplphysiol.00047.2019 30991014

[phy215208-bib-0052] Witlox, L. , Velthuis, M. J. , Boer, J. H. , Steins Bisschop, C. N. , Wall, E. V. , Meulen, W. J. , Schröder, C. D. , Peeters, P. H. , & May, A. M . (2019). Attendance and compliance with an exercise program during localized breast cancer treatment in a randomized controlled trial: The PACT study. PLoS One, 14(5), e0215517. 10.1371/journal.pone.0215517 31067223 PMC6505930

[phy215208-bib-0053] Zago, M. , Capodaglio, P. , Ferrario, C. , Tarabini, M. , & Galli, M. (2018). Whole‐body vibration training in obese subjects: A systematic review. PLoS One, 13(9), e0202866. 10.1371/journal.pone.0202866 30183742 PMC6124767

